# Microwave Plasma Pencil for Surface Treatment: Numerical Study of Electromagnetic Radiation and Experimental Verification

**DOI:** 10.3390/ma17174369

**Published:** 2024-09-04

**Authors:** Helena Nowakowska, Dariusz Czylkowski, Bartosz Hrycak, Mariusz Jasiński

**Affiliations:** Institute of Fluid Flow Machinery, Polish Academy of Sciences, Fiszera 14, 80-231 Gdansk, Poland; dczylkowski@imp.gda.pl (D.C.); bhrycak@imp.gda.pl (B.H.); mj@imp.gda.pl (M.J.)

**Keywords:** microwave plasma source, microwave plasma pencil, atmospheric pressure plasma, surface modification

## Abstract

An atmospheric pressure plasma source of the microwave plasma pencil type utilizing a coaxial line is presented. The generated plasma takes the form of a cylinder up to about 30 mm long and up to 5 mm in diameter. It is suitable for surface sterilization, surface treatment, and material processing. This study numerically analyzes the electromagnetic radiation emitted by the plasma pencil, which compromises performance and poses safety risks. Electric field distributions, radiation patterns, the ratio of the power entering the discharge to the incident wave power, and the ratio of radiated power to entering power were numerically investigated for different plasma parameters and pencil lengths. Results indicate that increasing electron density, gas temperature, plasma length, and pencil length increases the radiated power by up to more than 60% of the entering power, and the radiation patterns can be highly non-uniform with strong backward lobe. The numerical finding were qualitatively confirmed experimentally. It was also found that it is possible to reduce radiation from the device by using appropriately designed cones, the presence of which does not impede its performance.

## 1. Introduction

Plasma surface treatment and material processing include techniques that use ionized gases to modify, refine, and enhance the physical and chemical properties of materials in a variety of applications. The interaction between plasma species and the material surface allows for precise control of material properties, improving performance properties such as hardness, corrosion resistance, and electrical conductivity [1,2,3]. Utilizing microwave energy to generate plasma, these methods enable the treatment of a wide range of materials, such as metals, polymers, ceramics, and composites [4,5,6]. Unique features of microwave plasma include high electron densities, which increase reaction rates and processing efficiency, and the ability to maintain a low bulk gas temperature while keeping high electron temperatures, making microwave plasma suitable for treating heat-sensitive materials. In plasma-assisted chemical processes, microwave plasmas are used for advanced synthesis of nanomaterials, such as graphene [7], carbon, TiO_2_ or ZnO nanoparticles, nanotubes, and nanowires [8,9,10,11,12,13,14,15]. In the field of surface treatment, microwave plasmas offer a cost-effective and scalable methods for plasma etching, cleaning, deposition, and functionalization by enhancing properties such as adhesion, hydrophilicity, and biocompatibility [16,17,18,19,20,21]. They benefit industries such as electronics (semiconductors, thin-film transistors, and photovoltaic cells), optics, coatings, biomedical engineering, aerospace, automotive, and textiles and packaging [22,23,24,25].

Microwave plasmas can be produced in various structures, such as waveguides [8], coaxial lines [10], and strip lines [26]. They can be generated in dielectric tubes as plasma columns [27], flames [17], and sheets [28], or in open air as torches [7,11] and jets [29]. An overview of them, along with advantages and disadvantages, can be found, for example, in [30,31,32].

Surface treatment does not require a large volume of plasma, which is why plasma sheets or jets are most often used for this purpose. The latter is usually produced in microwave plasma sources (MPSs), whose design uses a coaxial line powered by a coaxial cable. This makes the device small, efficient, and portable, and requires relatively little power (less than 100 W, and often around 1 W). Such devices, designed in the 1990s, were called torches [33,34] and were used for atomic spectroscopy. A similar device, but for surface treatment applications, was designed in our laboratory. Its different versions and plasma properties have been described in previous articles [35,36]. We named this plasma source a microwave plasma pencil (MPP) due to the fact that the plasma produced is shaped like a thin elongated column, and the device can be operated by holding it in hand and moving it along the surface as if writing. For the same reason, the term ‘plasma pencil’ was used for a high-frequency device designed for surface processing [37] and high-voltage-induced discharge employed for killing of leukemia cells [38]. The portability of the device and ease of manipulation sets it apart from other similar plasma sources, an overview of which can be found in [39].

Our MPP was used to deactivate microorganisms such as bacteria and fungi [40]. It was also used for plasma-induced crystallization of TiO_2_ nanotubes in [41], where the anodic TiO_2_ nanotube samples were annealed in a nitrogen plasma flame for 30 s at a gas flow rate of 20 L/min and microwave power of 100 W. A photoresponse comparable to traditional furnace annealing, preserving the nanotubes’ structure, was obtained. The method showed good reproducibility and stability, making it a time- and energy-saving alternative to traditional furnace annealing and pressurized plasma chamber methods.

The main disadvantage of MPSs is that if they are designed as open-space structures and they are not shielded, they emit electromagnetic (EM) energy into the surrounding space. Our previous articles on the surfatron [42] and torch [43] showed that the value of the radiated power can reach up to 40% of the power delivered to the discharge. Radiation from an MPS can be used by various types of plasma antennas [44]. However, in most applications, it is an unfavorable phenomenon, as it has a harmful effect on people and equipment and makes the plasma source less energy-efficient. In order to limit or block the radiation from an MPS, metal shields can be used, which are usually in the form of tubes surrounding the plasma [42,45]. It is worth noting that the shielding elements do not have to be made of solid metal and do not have to cover the device completely. Almost complete shielding can be achieved even if the screen is a grid or has holes cut out (e.g., for observing plasma). It is important that the size of the holes in the shield is much smaller than the wavelength. Additionally, the shielding tube does not have to be completely closed. It is important that the wave cannot propagate in it. We demonstrated this experimentally for the case of a torch [43]. Estimating the radiated (and therefore wasted) power and finding a method to reduce it can improve the efficiency of the proposed device.

The purpose of this article is to numerically analyze the EM radiation emitted by the MPP with a coaxial line design. We perform an analysis of the relationship between incident and reflected wave power, and the power entering the device and power radiated from it. The analysis is parametric, i.e., the effect of plasma parameters (electron density, electron-neutral collision frequency, and plasma length) on the results is studied. The influence of adding shielding elements on radiated and reflected power is also investigated. Commercial software COMSOL Multiphysics is used for the calculations [46]. The obtained results are qualitatively evaluated experimentally.

## 2. Design and Properties of MPP

**Figure 1:**
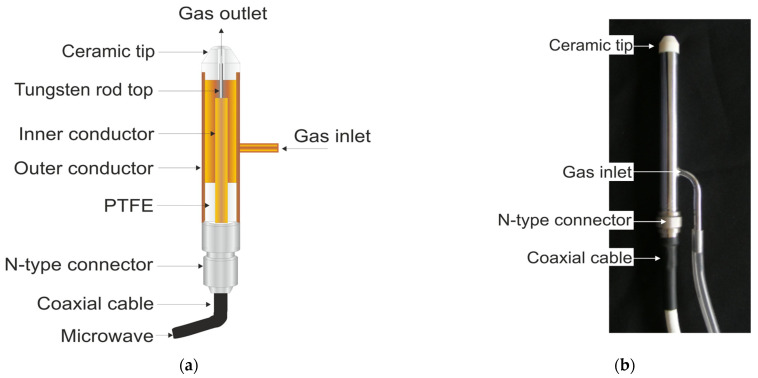
(**a**) Schematic from [36], modified by authors (reprinted with permission), and (**b**) photo of the analyzed MPP.

A schematic and photo of the analyzed MPP is shown in Figure 1. The outer conductor of the MPP is made of brass, and a pipe is attached to its wall through which the working gas is introduced. The inner conductor is also made of brass and has a tungsten rod (with a smaller diameter) at the end. It is fixed inside the outer conductor with a centering disc made of PTFE. The tungsten rod makes the device more resistant to damage from elevated temperature. The presented MPP differs from the previously described ones in that the inner conductor protrudes above the outer conductor by 5 mm. The working gas flows through a channel between the inner and outer conductors. The MPP is fed with microwaves at a frequency of *f* = 2.45 GHz through a 50 Ω coaxial cable using an N-type connector. The device can work stably with microwave power in the range 5–80 W and gas flow rate in the range 1–15 L min^−1^. The plasma is generated at the end of the tungsten rod and has a shape similar to a candle flame (in Ar and Ar/O_2_ mixtures at low entering powers) or a plasma jet (in Ar/O_2_ mixtures at high entering powers). For Ar, the plasma length is from 5 to 25 mm and its diameter is from 0.5 to 2 mm, while for Ar/O_2_, the plasma length is from 2 to 30 mm and the diameter is from 2 to 16 mm.

**Figure 2:**
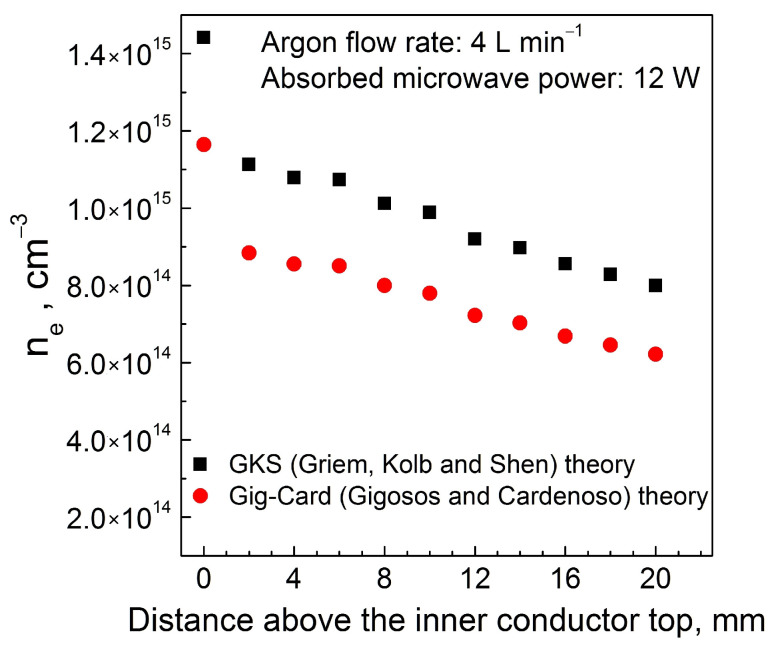
Electron density profile along the plasma determined in [36], modified by authors (reprinted with permission).

The plasma gas temperature was determined in [37] and the electron density in [36]. The gas temperature was measured at the end of the plasma and in its core. The temperature at the end was always lower (by about 100 °C) than the temperature in the core and its value ranged from 80 to 300 °C, depending on the operating conditions. Increasing the flow rate caused the temperature to decrease, increasing the absorbed power caused the temperature to increase, as did the addition of O_2_ to Ar. The distribution of the electron density along the plasma, determined in [36], is shown in Figure 2. The electron density determined using a GKS theory [47] is above 1.4 × 10^15^ cm^−3^ at the end of the rod, drops sharply to about 1.1 × 10^15^ cm^−3^ at a distance of 2 mm, and then decreases almost linearly to about 8 × 10^15^ cm^−3^ at a distance of 20 mm. The electron densities determined using Gig-Card [48] are lower, but all patterns have similar characteristics. Increasing the power delivered to the discharge increases the electron density values, whereas increasing the gas flow rate from 1 to 5 [L min^−1^] does not change the electron density values.

## 3. Model Description

### 3.1. Power Flow Analysis

**Figure 3:**
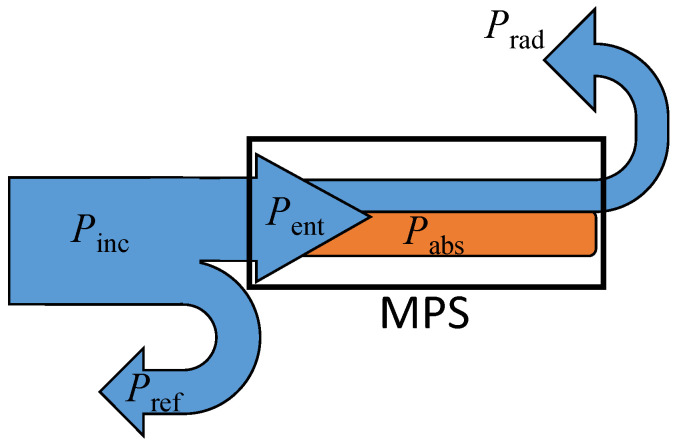
Power flow in an unshielded MPS.

A general scheme of power flow in an unshielded microwave plasma source can be found in our previous article [43]. Its simplified version is presented in Figure 3.

As is seen, the EM wave incident on the MPS (carrying the power *P*_inc_) partially reflects from it (*P*_ref_), and the rest enters the MPS (*P*_ent_). This power is partially radiated (*P*_rad_) and partially absorbed in the plasma (*P*_abs_). In this article, we ignore the power lost in the walls and other elements of the device’s construction. The power balance presented in Figure 3 can be written as follows:

*P*_inc_ = *P*_ent_ + *P*_ref_ = (*P*_rad_ + *P*_abs_) + *P*_ref_. (1)

The power *P*_inc_ is imposed in the calculation, whereas the other powers are calculated.

### 3.2. Calculation Domain

**Figure 4:**
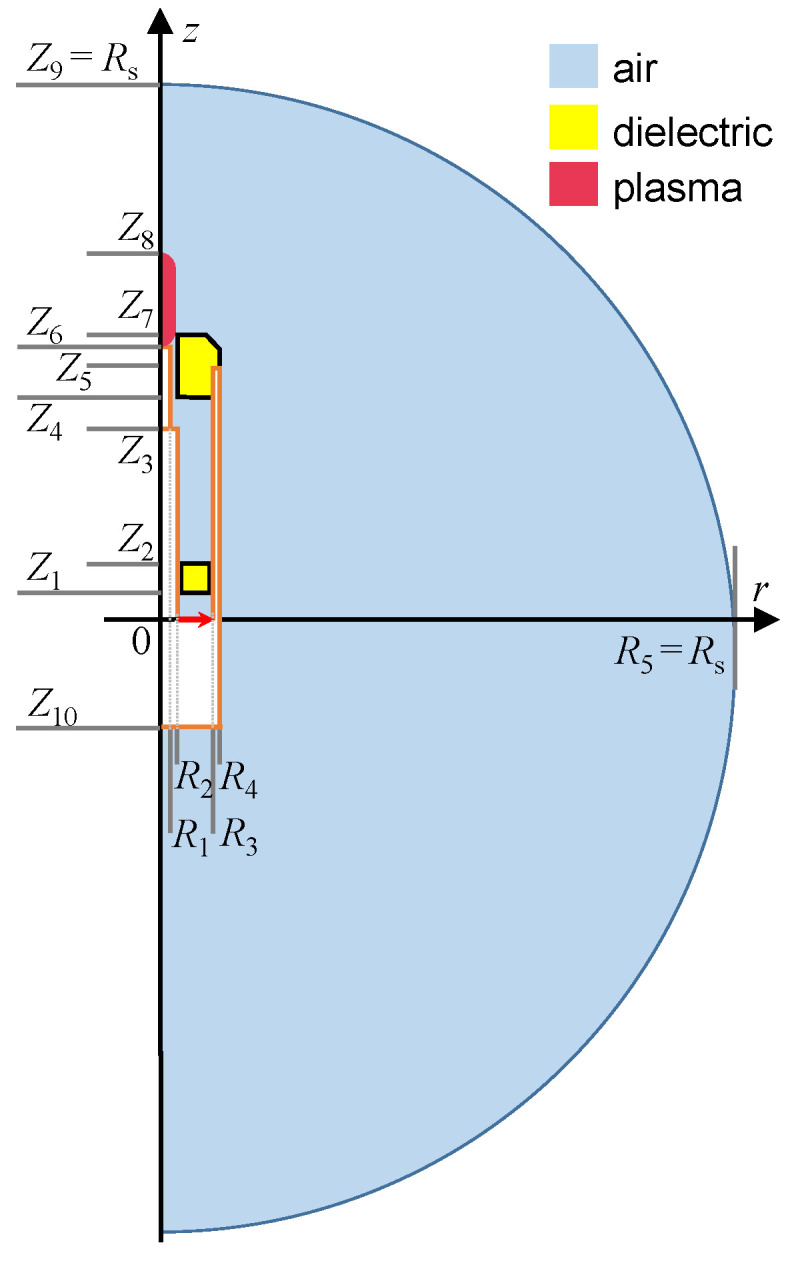
Schematic representation of the computational domain with geometry parameters in Table 1. Orange lines indicate metal surfaces. The red arrow indicates the excitation surface (drawing not to scale).

For the calculations, it is assumed that the metal gas supply pipe (shown in Figure 1) can be neglected, so that the system has axial symmetry, and the calculations can be performed in a two-dimensional, axisymmetric geometry. Figure 4 shows the numerical calculation domain in a cylindrical coordinate system, with radial and axial coordinates denoted as *r* and *z*, respectively. The MPP is placed in an air-filled sphere with a radius *R*_s_ much larger than the dimensions of the device. Such a sphere is called a radiation sphere in analysis of antenna properties. The dimensions of the device and plasma are listed in Table 1. The plasma radius is the same as the radius of the inner conductor *R*_2_. The plasma length *L*_p_ is a parameter of the calculations. The lower and upper sections of the plasma are rounded. Since the device is powered by a coaxial cable (shown in Figure 1), the length and bend of which can be changed, we also included its presence in the model. We designate the length of the cable below the excitation plane as *H*_d_, being a calculation parameter.

### 3.3. Governing Equations and Calculation Parameters

The main quantity necessary to analyze the phenomena occurring during microwave propagation and absorption in a plasma source is the electric field. Its spatial distribution can be found from the wave equation, which written in terms of a vector field phasor **E**(*r*, *z*) has the following form:

(2) $$\nabla \;\times \left(\nabla \;\times E\right)-k_{0}^{2}\varepsilon_{r}\mu_{r}\;E\;=0,$$

where ε_r_ is the complex relative permittivity and μ_r_ is the relative permeability of a medium (for all media μ_r_ = 1), *k*_0_ = ω/*c* is the free-space wavenumber, ω = 2π*f* is the angular field frequency, and *c* is the speed of light in free space. The relative permittivity of plasma ε_p_ is a complex quantity and can be found from the Drude–Lorentz formula [49]:

(3) $$\varepsilon_{p}=1-\frac{n}{1+s^{2}}-j\frac{ns}{1+s^{2}},$$

where *n* = *n*_e_/*n*_c_ and *s* = ν/ω are the normalized electron density and electron collision frequency, respectively; *n*_e_ is the electron density; ν is the electron-neutral collision frequency for momentum transfer; *n*_c_ = ω^2^ε_0_*m*/*e*^2^ is the critical electron density; ε_0_ is the free-space permittivity; *e* and *m* are the electron charge and charge, respectively; and *j* is the imaginary unit. Although an MPS (with plasma as a nonlinear medium) can generate EM waves at frequencies other than the excitation frequency [50,51], particularly higher harmonics [52], this effect is usually neglected in plasma modeling, because single-frequency microwave discharge models give results sufficiently consistent with experiments [53].

The power absorbed in volume V can be found from the following:

(4) $$P_{\mathrm{abs}}=\int_{V}(1/2)\;\sigma {\left|E\right|}^{2}\mathrm{dV},$$

where σ is the electrical conductivity of plasma and σ = ωε_0_ Im(ε_p_). The power radiated through the surface **A** (*P*_rad_) can be found as a surface integral [54]:

(5) Prad=∫A(1/2) ReE × H∗dA,

where **H** is the phasor of the magnetic field, which can be determined from **E** using the Maxwell equations. The asterisk indicates a conjugated number and operators Re and Im denote the real and imaginary part of a complex number, respectively. The integrand in Equation (5) represents the Poynting vector and its absolute value, *S*, expresses the surface power density carried by the EM waves.

The following boundary conditions (BCs) are assumed. All metallic surfaces are perfect conductors, that is, the electric field has only a normal component on them. The scattering BCs for a spherical wave (with center at coordinate system center) are imposed on the radiation sphere, which means that the surface of the sphere is transparent to this wave. The symmetry BC is assumed on the axis, which implies that the radial component of the electric field is zero. In the excitation plane (red arrow seen in Figure 4), which is the power input, an electric field with a radial component characteristic of a coaxial line (i.e., proportional to 1/*r*) is assumed. The formulas for applied BCs can be found in the software documentation [46,55]. The BCs are summarized in Table 2.

**Figure 5:**
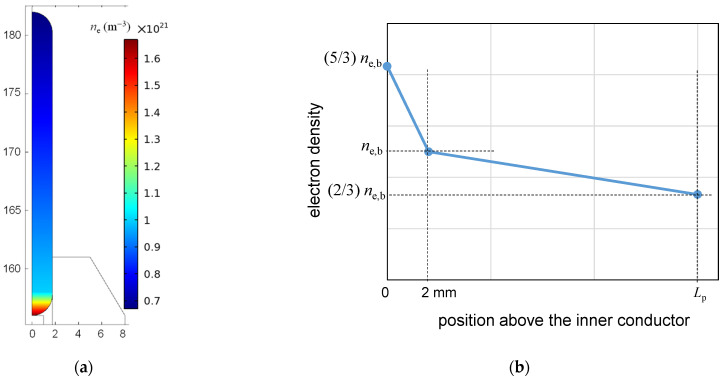
(**a**) Plasma column shape and a distribution of *n*_e_ in the plasma (for *n*_e,b_ = 1.5 × 10^21^ m^−3^); dimensions are in mm. (**b**) Assumed axial profiles of *n*_e_(*z*) along the plasma.

It is assumed that the plasma has the shape of a cylinder rounded at the bottom and top (see Figure 5a), the radial profile of electron density is constant, and the axial profile is similar to that presented in Figure 2, i.e., can be approximated by a broken line, with the breaking point at 2 mm above the inner conductor end. The parameter chosen to describe the profile is the electron density at the break point, *n*_e,b_, (it is shown in Figure 5b). The maximal value of the electron density is (5/3) *n*_e,b_, and the minimal value is (2/3) *n*_e,b_. The general shape of this distribution was preserved in calculations, only its parameters, namely, *n*_e,b_ and the plasma length *L*_p_, were changed.

The electron-neutral collision frequency ν necessary to determine the electrical permittivity from Equation (3) is a quantity that depends on the gas nature and, within the framework of the classical collision theory, can be determined from the following equation:

ν(*w*) = *N Q*_c_(*w*) *w*, (6)

where *N* = *p*/*k*_B_*T* is the concentration of gas particles, *Q*_c_ is the electron cross-section for momentum transfer, *w* is the electron velocity, *p* and *T* are the pressure and temperature of the gas, respectively, and *k*_B_ is the Boltzmann constant. The collision frequency increases with the increase in the velocity (and therefore energy) of electrons and decreases with the increase in gas temperature. To estimate the frequency ν value, we did not use the simplified formula (Equation (6)) (used here to show general relationships), but rather the BOLSIG+ program (ver. 11/2019) described in [56], along with cross-sections from [57], with the electron temperature equal to 1 Ev and the gas temperature from 300 to 600 K. For the calculations, it is assumed that the parameter *s* = ν/ω is constant in the plasma column and its value varies in the range from 10 to 100 (with smaller values corresponding to higher gas temperatures). For all calculations, the incident wave power is equal to 10 W. The choice of this value does not affect the reported results, since they are presented as relative relationships and Equation (2) is linear with respect to **E**.

## 4. Calculation Results

All the calculations were performed for the field frequency *f* = ω/2π = 2.45 GHz, the dimensions shown in Table 1, and the dielectric relative electric permittivity ε_d_ = 2.05, using a commercial software COMSOL Multiphysic^®^ with RF Module ver. 4.1 [55]. This software is an advanced tool that uses the finite element method (FEM) [58] to model complex physical problems, with robust solvers and meshing algorithms. It is also used for modeling phenomena involving microwave plasma and surface treatment [16,24,59,60]. Calculations were performed for default settings (relative error 0.001 and stationary MUMPS solver). Full documentation is available on the company website [46].

### 4.1. Spatial Distribution of the Electric Field and Radiation Pattern

**Figure 6:**
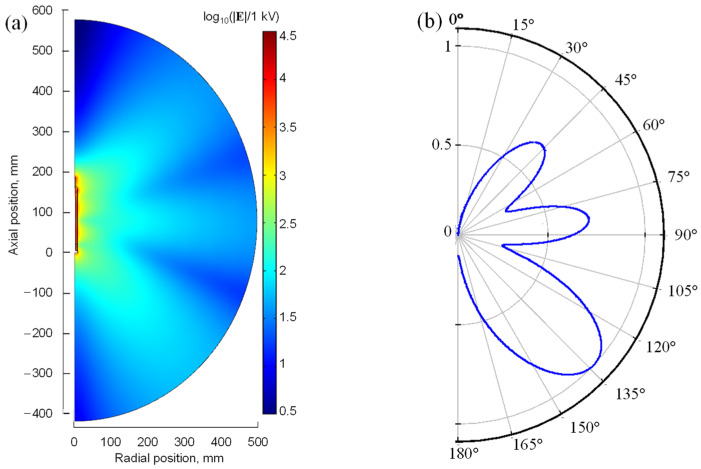
(**a**) Calculated spatial distribution of the electric field strength |**E**|. (**b**) Calculated normalized electric field pattern. Results for the power *P*_inc_ = 10 W (*P*_ent_ = 9.46 W), plasma length *L*_p_ = 22.5 mm, electron density *n*_e,b_ = 1 × 10^21^ m^−3^, *s* = 20, *H*_d_ = 5 mm, and *R_s_* = 500 mm.

The solution to Equation (2) is the spatial distribution of the electric field **E**. Figure 6a shows the field strength |**E**| inside the radiation sphere surrounding the MPP for the power *P*_inc_ = 10 W (*P*_ent_ = 9.46 W), electron density at the break point *n*_e,b_ = 1 × 10^21^ m^−3^, collision frequency *s* = 20, plasma length *L*_p_ = 22.5 mm, *H*_d_ = 5 mm, and sphere radius *R*_s_ = 500 mm. This plasma is quite long and can be obtained for high power absorption and a high gas flow rate in argon. Figure 6b shows the radiation pattern as it is typically presented for antennas [54]. It shows the value of the electric field (normalized to its maximum value) on the surface of the radiation sphere in the polar system for a fixed *r-z* plane. As can be seen from Figure 6a it the highest values of |**E**| are inside the coaxial line and near the plasma. A standing wave pattern is in the vicinity of the outer metal walls of the MPP. With increasing distance from the center of the coordinate system, the electric field strength decreases. From Figure 6b, it is seen that on the surface of the radiation sphere, the electric field is not uniform. Its value changes with the polar angle θ, which is defined as follows:

θ = atan(*r*/*z*), (7)

Along the *z*-axis (θ = 0° and θ = 180°), |**E**| values are zero. The other minima are for θ ≅ 60° and θ ≅ 105°. Three maxima of |**E**| are seen for θ ≅ 45°, θ ≅ 80°, and θ ≅ 135°. The features of the field distributions determined for other electron densities *n*_e,b_ are very similar although the values differ.

### 4.2. The Effect of the Radiation Sphere Radius and Electron Density on Power Ratios

**Figure 7:**
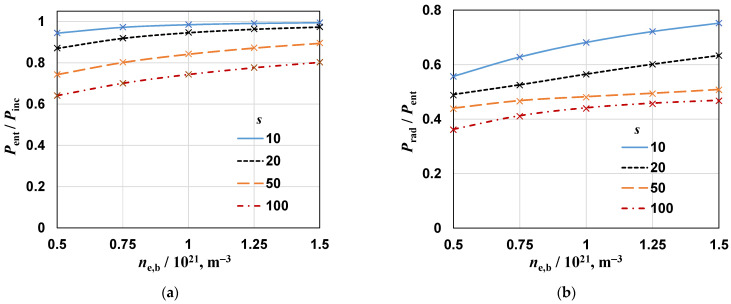
The effect of the electron density *n*_e,b_ on power ratios for different values of *s*: (**a**) *P*_ent_/*P*_inc_ and (**b**) *P*_rad_/*P*_ent_; plasma length *L*_p_ = 22.5 mm, *H*_d_ = 5 mm; Lines: *R*_s_ = 500 mm; crosses *R*_s_ = 1000 mm.

From the spatial distributions of the electric field, it is possible to determine the other quantities describing the EM field, including the radiated and absorbed powers. Figure 7 shows the influence of the density *n*_e,b_ on the power ratios for four values of *s*, two values of *R*_s_, plasma length *L*_p_ = 22.5 mm, and *H*_d_ = 5 mm; Figure 7a,b show the ratio *P*_ent_/*P*_inc_ and *P*_rad_/*P*_ent_, respectively. As one can see, the results obtained for different values of *R*_s_ practically do not differ. This means that for calculations, it is enough to take a sphere with a smaller radius, which reduces the calculation time. The *P*_ent_/*P*_inc_ ratio is a quantity that describes the efficiency of power transfer from the microwave generator to the device, i.e., it characterizes matching of the device to the power line. The matching is perfect when this ratio is 1. It is seen that the *P*_ent_/*P*_inc_ ratio increases with *n*_e,b_ and decreases with increasing *s* (i.e., with increasing gas temperature, see Equation (6)). All values are greater than 0.6, which means that the device is quite well matched to the feeding line. It should be noted that in experimental conditions, it is always possible to match the device by using appropriate tuners. The *P*_rad_/*P*_ent_ ratio increases with increasing electron *n*_e,b_ and decreases with increasing *s* with a minimal value of 0.35 and maximum value of 0.75. These are very high values. They mean that at higher power (note that the electron density and gas temperature increase with power), radiation losses can reach up to 75% of the entering power.

### 4.3. The Effect of the Plasma Column Length L_p_ on Power Ratios

**Figure 8:**
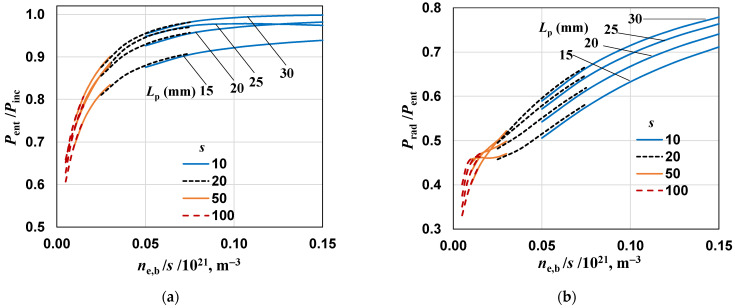
The effect of the normalized electron density *n*_e,b_/*s* on power ratios for different values of *s* and *L*_p_. (**a**) *P*_ent_/*P*_inc_ and (**b**) *P*_rad_/*P*_ent_; *H*_d_ = 5 mm, *R*_s_ = 500 mm.

The effect of plasma length on the ratios *P*_ent_/*P*_inc_ and *P*_rad_/*P*_ent_ is shown in Figure 8. For the analyzed discharge, *s* >> 1 occurs, so according to Equation (3): Re(|ε_p_|) << Im(|ε_p_|) and Im(ε_p_)~*n*/*s*. This means that the ratio *n*/*s* is a good parameter for presenting the calculation results and therefore it was used in Figure 8 and following. Comparing Figure 7a and Figure 8a, as well as Figure 7b and Figure 8b, it can be seen that using in such a presentation, we practically obtain one continuous curve for a given *L*_p_ and different values of *s*. The conclusions from Figure 8 essentially confirm those from Figure 7. Additionally, it can be seen that increasing the plasma length generally causes an increase in both ratios (except for *P*_ent_/*P*_inc_ for *L*_p_ = 25 mm and *n*_e,b_/*s* > 0.12 as well as for *P*_rad_/*P*_ent_ and *n*_e,b_/*s* < 0.02). From Figure 8a, it is also seen that if *n*_e,b_ decreases, the *P*_ent_/*P*_inc_ ratio strongly decreases. We determined that for conditions such as in Figure 6 and *n*_e,b_ = 0 (the case when there is no plasma), this ratio is about 0.07. This means that the power of the reflected wave is about 93% of the power of the incident wave. In addition, since the power is not absorbed, all the entering power is radiated from the device.

### 4.4. The Effect of the MPP Length on Power Ratios and Radiation Patterns

**Figure 9:**
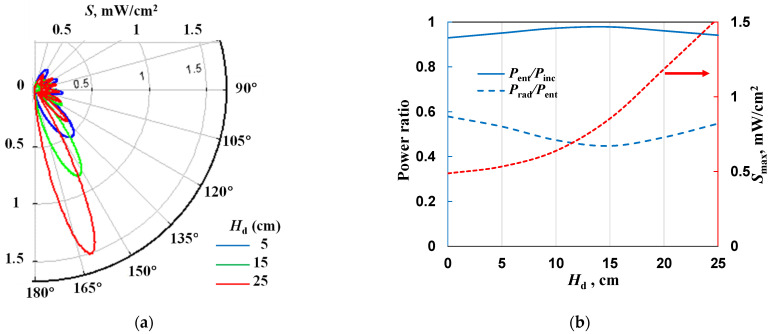
The effect of the *H*_d_ on (**a**) radiation patterns and (**b**) *P*_ent_/*P*_inc_, *P*_rad_/*P*_ent_, and *S*_max_ for *s* = 20, *n*_e_ = 10^21^ m^−3^, *L*_p_ = 25 mm, *P*_ent_ = 10 W, *R*_s_ = 500 mm.

Our MPP is fed from a coaxial line connected axially (see Figure 1b) (in other designs, it is connected perpendicularly), so we investigated the effect of the length of the power line (by changing the value of *H*_d_) on EM field distributions and *P*_ent_/*P*_inc_ and *P*_rad_/*P*_ent_. From Figure 9a, it can be seen that the extension of the power line causes the main lobe to elongate, and its direction shifts backward, i.e., toward larger polar angles. (This figure, but with a different scale, can be found in the Supplementary Materials). The increase in maximum power density *S*_max_ with increasing line length is also seen in Figure 9b. It can be seen, also, that the total radiated power weakly depends on *H*_d_, as does the power of the reflected wave.

The results obtained are important because they show that when studying the EM field around an MPS, one should always analyze the entire surrounding. For our MPP, the radiation is directed backward, toward the person using the device, which is an unfavorable phenomenon.

From Figure 7, Figure 8 and Figure 9, it can be seen that the value of radiated power increases with increasing electron density, gas temperature, and plasma length and can reach a value of 80% of the entering power. This is a very high value. These calculations show that the power entering the discharge is not absorbed but lost. However, under experimental conditions, an increase in input power will not necessarily result in an extension of the plasma or an increase in electron density and gas temperature, because the increase in power will be lost to radiation. Due to the rather low powers used in the MPP, the risk to personnel is not necessarily high, but the equipment should be as energy efficient as possible.

### 4.5. The Effect of the Shielding Cone on Power Ratios and Radiation Patterns

**Figure 10:**
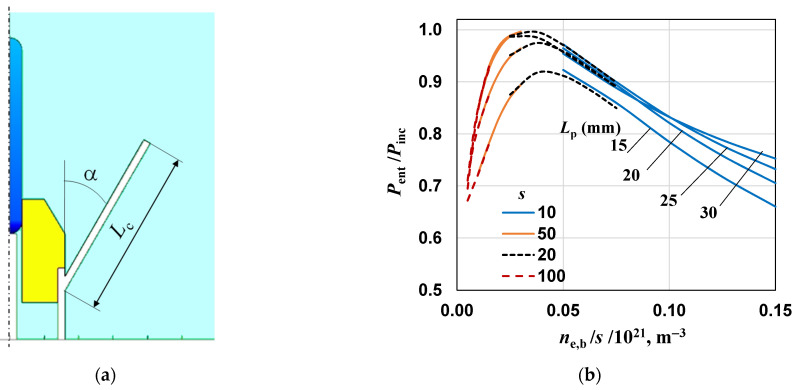
(**a**) Position and dimensions of the shielding cone. (**b**) The effect of the electron density *n*_e,b_/*s* on *P*_ent_/*P*_inc_ ratio for cone 1 (α = 30° and *L*_c_ = 20 mm).

To protect personnel and equipment and increase efficiency, shielding elements in the form of metal shielding tubes are usually used. In order to maintain the usability of the device (its portability, the ability to work with one hand) and not to limit the possibilities of its use, we propose the use of cones instead of shielding tubes, which partially limit the plasma flame, allowing for the plasma to access the treated surface. The method of placing the cone and determining its geometry is shown in Figure 10a.

We performed the calculations for several cones with different angles α and lengths *L*_c_. The value of the *P*_ent_/*P*_inc_ ratio for cone 1 (angle α = 30° and *L*_c_ = 20 mm) and different plasma lengths is shown in Figure 10b. As can be seen, the ratio is greater than 0.6 under all conditions and, compared to the unshielded case seen in Figure 8a, has a maximum for *n*_e,b_/*s* ≅ 2.5 × 10^19^ m^−3^. The results for cone 2 (α = 20° and *L*_c_ = 25 mm) are similar to that, only the lines are more closely spaced (we do not show them in a figure). As mentioned earlier, these dependencies are not very important under experimental conditions because reflections and mismatches in the microwave power line can be compensated by using tuners. However, they do show that when a cone is added, the device should be re-tuned.

**Figure 11:**
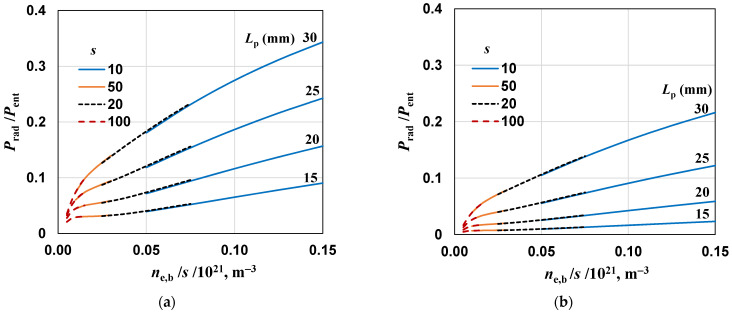
The same as in Figure 8b but for (**a**) cone 1 (α = 30° and *L*_c_ = 20 mm) and (**b**) cone 2 (α = 20° and *L*_c_ = 25 mm).

What is important are the radiated powers expressed as *P*_rad_/*P*_ent_, shown in Figure 11 for both cones. Comparing with the results for the unshielded device (Figure 8b), it can be seen that the maximum radiated power is more than twice less for cone 1 and almost four times less for cone 2. As expected, the radiation is lower for a cone with a smaller opening angle and longer length.

**Figure 12:**
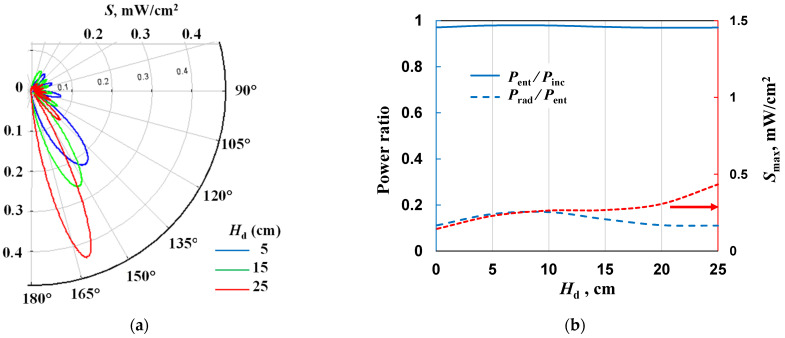
The effect of the *H*_d_ on (**a**) radiation patterns and (**b**) *P*_ent_/*P*_inc_, *P*_rad_/*P*_ent_, and *S*_max_ for *s* = 20, *n*_e_ = 10^21^ m^−3^, *L*_p_ = 25 mm, *P*_ent_ = 10 W, *R*_s_ = 500 mm and cone 3 (α = 20° and *L*_c_ = 20 mm).

In the next step of the calculation, we checked how the length of the power line *H*_d_ affects the radiation patterns and radiated power when a shielding cone is used. As can be seen from Figure 12a, when increasing the length, the main lobe shifts backward and the maximum radiated power increases. (This figure, but with a different scale, can be found in the Supplementary Materials). From Figure 12b, it can be seen that the length of *H*_d_ weakly affects the entering and radiated powers. These relationships follow a similar pattern to those shown in Figure 9b, only the values are different.

## 5. Experimental Results and Discussion

### 5.1. Experimental Setup

A direct comparison of modeling and experimental results is not possible, because the assumptions made for calculations are impossible to reproduce in laboratory conditions. Under ideal conditions, radiated power values and radiation patterns are measured in an anechoic chamber without the presence of additional elements that could disturb the EM field distribution. Our MPP is designed for use in the laboratory, in the presence of equipment, treated surface and personnel, which can change their position. In addition, the length and diameter of the plasma, temperature, and electron density distributions should be measured each time, since the power radiated by the device depends on all these parameters. What is more, the mere presence of the measuring device, which changes position with the operator’s hand, distorts the results. Therefore, our goal was only to qualitatively compare the behavior of the MPP without shielding (configuration 1) and with shielding (configuration 2). For the experiments, we chose cone 3 with an opening angle of 20° and a length of 20 mm.

**Figure 13:**
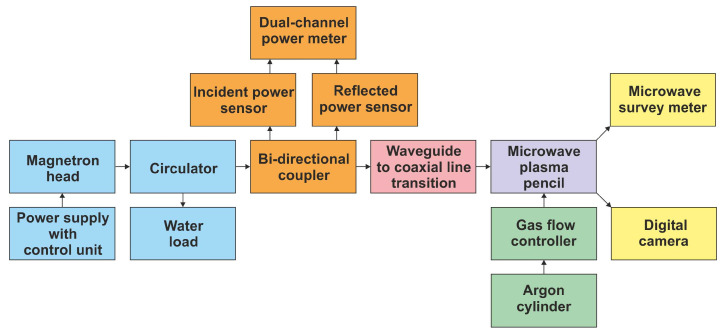
The diagram of the setup for the experimental verification of the numerical analysis. (background changed to a lighter color).

The diagram of the experimental setup is shown in Figure 13. A microwave generator (GMP 20 KE/D, Sairem, Décines-Charpieu, France), with control unit and a magnetron head operated at a microwave frequency of 2.45 GHz in continuous mode was used. To protect the magnetron head from excessive reflected power, a water-cooled circulator was utilized. A calibrated two-way coupler with incident and reflected power sensors (E9301A, Agilent, Santa Clara, CA, USA) and a dual-channel digital power meter (E4419B, Agilent, Santa Clara, CA, USA) were employed to monitor incident and reflected microwave power levels. Since a standard WR 340 rectangular waveguide was used to transmit power from the microwave generator, a transition from the waveguide to a coaxial line was used to allow for the MPP to be further fed from a 50 Ω dielectric-filled coaxial line. There is no tuning element in the setup. It can be added (e.g., three-stub tuner) during regular operation to minimize the reflected wave power. Argon (≥99.995% vol.) was a plasma-forming gas, with its flow regulated by a mass flow controller (F-201AC-FAB-33-V, Bronkhorst, Ruurlo, The Netherlands). The structure of the MPP was investigated with a digital camera (EOS 550D, Canon, Tokyo, Japan). The radiated power density was measured with a microwave survey meter (HI-1600, ETS-Lindgren, Cedar Park, TX, USA), calibrated from 1 to 10 mW/cm^2^ at a frequency of 2.45 GHz.

### 5.2. Experimental Results

**Figure 14:**
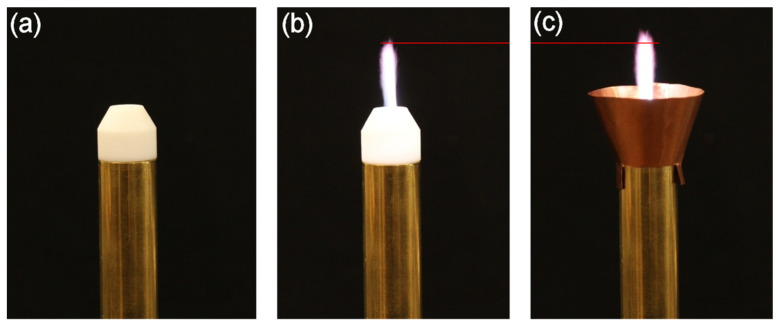
Photos of the MPP at different configurations: (**a**) unshielded without plasma, (**b**) unshielded with plasma (configuration 1), (**c**) with a cone-shaped screen and plasma (configuration 2). Argon flow rate is 10 L min^−1^. Entering power (*P*_ent_) is 50 W. The red horizontal lines are drawn at the same height to show the differences in flame length.

Figure 14 presents photographs of the MPP for three cases: the unshielded MPP without plasma, the unshielded MPP with plasma (configuration 1), and the MPP with a cone-shaped screen (*L*_c_ = 20 mm, opening angle of 20°) with plasma (configuration 2). The gas flow rate was 10 L min^−1^ and entering power (*P*_ent_) was 50 W. The cone was made of thin copper sheet. Its lower section adheres to the outer conductor of the coaxial line and is in contact with the ceramic tip. Making and fixing such an item is very easy. By comparing Figure 14b with Figure 14c, it can be seen that the addition of the cone-shaped screen causes a small increase in the width and length (from about 23 mm to about 25 mm) of the plasma flame. Since the entering power is the same, we attribute this effect to the reduction in radiated power (and hence increase in absorbed power) after the cone is applied.

**Figure 15:**
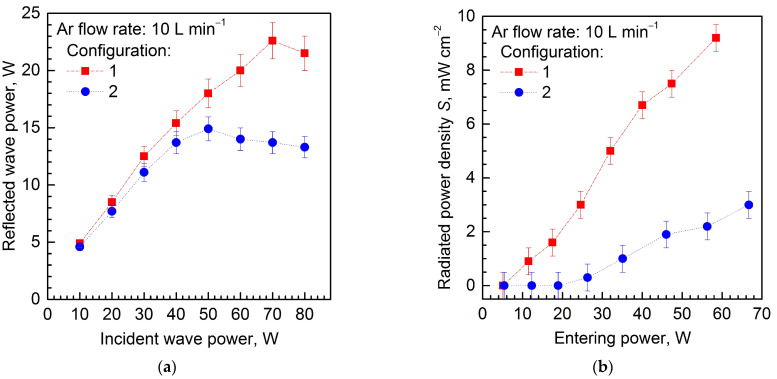
(**a**) Reflected wave power (*P*_ref_) versus incident wave power (*P*_inc_) for two configurations of the MPP; (**b**) radiated power density (*S*) versus entering power (*P*_ent_) for two configurations of the MPP measured at a distance of 90 mm from the inner conductor top at an angle of 135°.

Figure 15a illustrates the effect of the incident wave power (*P*_inc_) on the reflected wave power (*P*_ref_) for two configurations of the MPP and the gas flow rate 10 L min^−1^. As can be seen, using shielding cone (configuration 2) results in a reduction in reflected wave power over the whole range of the incident wave power (10–80 W). Table 3 presents the values of the parameters used in the experiment.

There is no straightforward translation of experimental results into numerical results, since the plasma parameters were not measured. However, it is clear from Figure 8a and Figure 10b that the presence of the cone changes the *P*_ent_/*P*_inc_ (= 1 – *P*_rad_/*P*_inc_) ratio, that is, it changes the degree of matching between the device and the feeding line. The same conclusions can be drawn from comparing the experimental curves in Figure 15a.

Figure 15b presents the experimental results on the dependence of the measured radiated power density (*S*) on the entering power (*P*_ent_). The radiated power density was measured at a distance of 90 mm from the inner conductor top at an angle of 135°. As expected, an almost linear increase in radiated power density values with increasing input power for both cases is seen. For configuration 2, the power density is about 3.5 times lower than for configuration 1 (at *P*_ent_ = 58 W), which means that in this direction, the radiation is significantly reduced. However, as we know from calculations, the radiation patterns are strongly non-uniform; therefore, we measured the power density for different angles.

**Figure 16:**
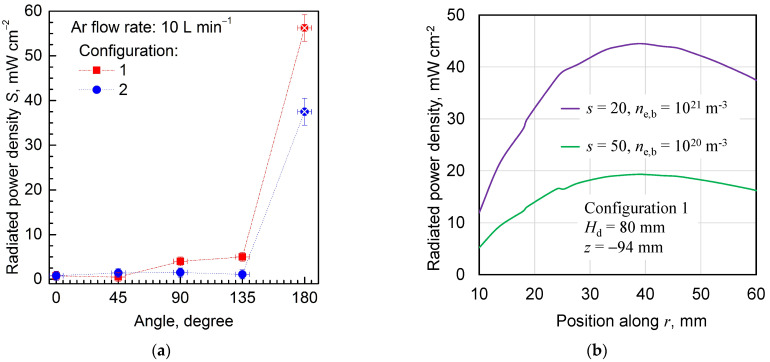
Radiated power density (*S*) for *P*_ent_ = 50 W (**a**) measured at different angles for two configurations of the MPP; distance of 100 mm from the inner conductor top (explanation about value for 180° angle—see in the text), (**b**) calculated for configuration 1, *H*_b_ = 8 cm, *z* = −94 mm, and two sets of plasma parameters.

Figure 16a shows the radiated power density (*S*) measured at different angles from the longitudinal axis of the MPP for the entering power (*P*_ent_) equal to 50 W. In that case, the radiated power density was measured at a distance of 100 mm from the inner conductor top (except for the 180° angle for which the distance was 250 mm). Since for the angle of 180° and the distance of 100 mm the value of radiation density was off the scale of our meter, we measured it for a distance of 250 mm. Measured radiated powers were 9 mWcm^−2^ and 6 mWcm^−2^ for configuration 1 and configuration 2, respectively. Due to the fact that the power density decreases approximately with the square of the distance, we multiplied the result times (250/100)^2^ = 6.25. Such a result is represented by the points marked with an additional cross. As can be seen, the values of the radiated power density for angles of 0° and 45° are close to each other. For angles of 90°, 135°, and 180°, the radiation for configuration 2 is smaller than for configuration 1. For an angle of 135°, the radiated power density value was equal to 5 mW cm^−2^ for configuration 1 and to 1.1 mW cm^−2^ for configuration 2, which means that the radiation in this direction is about 4.5 times larger. For an angle of 180°, this ratio is about 1.5. Experimental results confirm that MPP radiates microwave power into space. The radiated power increases almost linearly with the entering power. The radiation patterns are inhomogeneous, and the main lobe is directed backward. The use of a shielding cone reduces the radiation, with the main lobe pointing backward anyway. This effect is due to the presence of the coaxial line, which is the conductor along which the EM standing wave is excited. Note that this backward radiation is not a leakage of energy from the connector (as one would expect). Figure 16b shows the values of *S* calculated for two sets of plasma parameters and an example length *H*_d_ = 80 mm along a 50 mm long section located at a height of *z* = –94 mm, i.e., 250 mm below the level of the inner conductor top. As can be seen, the values of *S* depend strongly on the position (as seen also from Figure 8, where the lobes are very narrow) and on the plasma parameters. However, the results are of the same order as those obtained experimentally (*S* = 9 mWcm^−2^ for configuration 1). The fine inhomogeneities observed along the line are a normal effect for the results obtained by the FEM.

## 6. Conclusions

We carried out numerical calculations to determine the EM field distributions, radiated power, and efficiency of microwave power transfer to the plasma for an MPP and typical plasma parameters. Calculations show that the radiated power increases with increasing electron density, gas temperature, and plasma length, which is associated with an increase in absorbed power. At the highest experimentally possible parameters, the value of radiated power can reach 80% of the power entering the discharge. The main lobe of the radiation pattern is directed backward. Lengthening the power line increases the value of this lobe and moves it further backward.

To reduce radiated power, one should use shielding elements. In order not to interfere with the operation of the device, we suggest using metal cones. We investigated the effect of the shape of the shielding cone on the value of radiated power. The 20 mm long cone can reduce radiation losses by up to four times.

When interpreting the results, it is important to remember that the presented model is a parametric study and not a self-consistent model. Under experimental conditions, negative feedback can be expected, i.e., that with an increase in the incident wave power (and the power entering the discharge), the radiated power will increase, and therefore the power absorbed in the plasma will grow weakly or will be constant.

The power used in the device is in the order of 50 W, which is much less than that used in a surfatron (up to 300 W) or torch (on the order of 1 kW), so the radiated power is also much less than that of these devices. This means that radiation damage to people and equipment will be lower. Nevertheless, reducing this power is important for improving the device’s efficiency and user safety.

## Figures and Tables

**Table 1:** Values of the geometry parameters presented in Figure 4. The quantities *R*_s_ (radiation sphere radius), *L*_p_ (plasma length), and *H*_d_ (length of the cable below the excitation plane) are parameters changed in the calculation.

Parameter	Description	Value, mm
*Z*_1_	Dielectric disk bottom edge	10
*Z*_2_	Dielectric disk upper edge	16
*Z*_3_	Top rod beginning	135
*Z*_4_	Ceramic tip bottom edge	146
*Z*_5_	Outer conductor end	151
*Z*_6_	Top rod end	156
*Z*_7_	Ceramic tip upper edge	161
*Z*_8_	Plasma column end	151 + *L*_p_
*Z*_9_	Radiation sphere radius	*R*_s_
*Z*_10_	MPP’s bottom edge	*H*_d_
*R*_1_	Top rod radius	1.1
*R*_2_	Inner conductor radius	1.75
*R*_3_	Outer conductor inner radius	7
*R*_4_	Outer conductor outer radius	8
*R*_5_	Radiation sphere radius	*R*_s_

**Table 2:** Boundary conditions for the electromagnetic field.

Boundary	Type	Formula
Metal surface	Perfect electric conductor	**n** × **E** = 0 ^1^
Axis	Axial symmetry	**n** × **E** = 0
Radiation sphere	Scattering boundary condition	**n** × (∇ × **E**) – *j k*_0_ **n** × (**E** × **n**) = 0
Excitation plane	Coaxial port with power ON	see [46,55]

^1^ **n** is a vector normal to the surface.

**Table 3:** Experimental parameters for two configurations of the MPP.

Configuration	Shielding	Argon Flow Rate, L min^−1^	Incident Wave Power, W
1	No shielding	10	10–80
2	Cone-shaped screen: *L*c = 20 mm, opening angle of 20°	10	10–80

## Data Availability

The raw data supporting the conclusions of this article will be made available by the authors on request.

## Supplementary Materials

The following supporting information can be downloaded at: https://www.mdpi.com/article/10.3390/ma17174369/s1, Figure S1: The effect of the *H*_d_ on (a) radiation patterns (no cone); Figure S2: The effect of the *H*_d_ on (a) radiation patterns (with cone).

## References

[1] Hegemann D., Gaiser S. (2021). Plasma Surface Engineering for Manmade Soft Materials: A Review. J. Phys. D Appl. Phys..

[2] Bertin M., Leitao E.M., Bickerton S., Verbeek C.J.R. (2024). A Review of Polymer Surface Modification by Cold Plasmas toward Bulk Functionalization. Plasma Process. Polym..

[3] Elashry S., ELsaeed H., El-Siragy N.M. (2022). Microwave Plasma Discharge-Assisted Surface Modification of PVA Films: Coatings and Food Packaging. Eur. Phys. J. Plus.

[4] Hnilica J., Potočňáková L., Stupavská M., Kudrle V. (2014). Rapid Surface Treatment of Polyamide 12 by Microwave Plasma Jet. Appl. Surf. Sci..

[5] Storr B., Kodali D., Chakrabarty K., Baker P.A., Rangari V., Catledge S.A. (2021). Single-Step Synthesis Process for High-Entropy Transition Metal Boride Powders Using Microwave Plasma. Ceramics.

[6] Munir M.A., Naz M.Y., Shukrullah S., Naz A., Irfan M., Rahman S., Mursal S.N.F. (2024). Tailoring Properties of Ni_0.50_Co_0.50_Dy_x_Fe_2−x_O_4_ Ceramics Using Microwave Non-Thermal Plasma for High-Frequency Devices. Appl. Phys. A.

[7] Jašek O., Toman J., Jurmanová J., Šnírer M., Kudrle V., Buršíková V. (2020). Study of Graphene Layer Growth on Dielectric Substrate in Microwave Plasma Torch at Atmospheric Pressure. Diam. Relat. Mater..

[8] Bundaleska N., Tsyganov D., Dias A., Felizardo E., Henriques J., Dias F.M., Abrashev M., Kissovski J., Tatarova E. (2018). Microwave Plasma Enabled Synthesis of Free Standing Carbon Nanostructures at Atmospheric Pressure Conditions. Phys. Chem. Chem. Phys..

[9] Lee B.-J., Jo S.-I., Jeong G.-H. (2019). Synthesis and in Situ Nitrogen Doping of ZnO Nanomaterials Using a Microwave Plasma System at Atmospheric Pressure. Appl. Phys. A.

[10] Li D., Tong L., Gao B. (2020). Synthesis of Multiwalled Carbon Nanotubes on Stainless Steel by Atmospheric Pressure Microwave Plasma Chemical Vapor Deposition. Appl. Sci..

[11] Melero C., Rincón R., Muñoz J., Zhang G., Sun S., Perez A., Royuela O., González-Gago C., Calzada M.D. (2018). Scalable Graphene Production from Ethanol Decomposition by Microwave Argon Plasma Torch. Plasma Phys. Control Fusion.

[12] Heo S., Lim T., Kim B.S., Suk J.W., Bak M.S. (2022). Impact of N2 Admixture on the Synthesis of Graphitic Carbon Nanoparticles Using Atmospheric-Pressure Microwave Plasma. J. Phys. D Appl. Phys..

[13] Ouaras K., Lombardi G., Hassouni K. (2024). Nanoparticles Synthesis in Microwave Plasmas: Peculiarities and Comprehensive Insight. Sci. Rep..

[14] Dytrych P., Kluson P., Solcova O., Kment S., Stranak V., Cada M., Hubicka Z. (2015). Shape Selective Photoinduced Electrochemical Behavior of Thin ZnO Layers Prepared by Surfatron. Thin Solid Film..

[15] Kment S., Kluson P., Stranak V., Virostko P., Krysa J., Cada M., Pracharova J., Kohout M., Morozova M., Adamek P. (2009). Photo-Induced Electrochemical Functionality of the TiO_2_ Nanoscale Films. Electrochim. Acta.

[16] Narimisa M., Krčma F., Onyshchenko Y., Kozáková Z., Morent R., De Geyter N. (2020). Atmospheric Pressure Microwave Plasma Jet for Organic Thin Film Deposition. Polymers.

[17] Gazal Y., Dublanche-Tixier C., Chazelas C., Colas M., Carles P., Tristant P. (2016). Multi-Structural TiO_2_ Film Synthesised by an Atmospheric Pressure Plasma-Enhanced Chemical Vapour Deposition Microwave Torch. Thin Solid Film..

[18] Perraudeau A., Dublanche-Tixier C., Tristant P., Chazelas C. (2019). Dynamic Mode Optimization for the Deposition of Homogeneous TiO_2_ Thin Film by Atmospheric Pressure PECVD Using a Microwave Plasma Torch. Appl. Surf. Sci..

[19] Bónová L., Zhu W., Patel D.K., Krogstad D.V., Ruzic D.N. (2020). Atmospheric Pressure Microwave Plasma for Aluminum Surface Cleaning. J. Vac. Sci. Technol. A.

[20] Matsubayashi T., Hidaka H., Muguruma H. (2016). Microwave-Assisted Atmospheric Pressure Plasma Polymerization of Hexamethyldisiloxane. Jpn. J. Appl. Phys..

[21] Durocher-Jean A., Durán I.R., Asadollahi S., Laroche G., Stafford L. (2020). Deposition of Anti-Fog Coatings on Glass Substrates Using the Jet of an Open-to-Air Microwave Argon Plasma at Atmospheric Pressure. Plasma Process. Polym..

[22] Tudoran C., Roşu M.-C., Coroş M. (2020). A Concise Overview on Plasma Treatment for Application on Textile and Leather Materials. Plasma Process. Polym..

[23] Schnabel U., Andrasch M., Weltmann K.-D., Ehlbeck J., Schnabel U., Andrasch M. (2015). Inactivation of Microorganisms in Tyvek^®^ Packaging by Microwave Plasma Processed Air. Glob. J. Biol. Agric. Health Sci..

[24] Dong Y., Zhang J., Zhang P., Tian H., Yu D. (2022). Jet Morphology Analysis of Microwave-Generated Plasma for Microfabrication of Optics. Plasma Process. Polym..

[25] Li Y., Bai Y., Yu D., Wang R., Mu Y., Jin W., Yu B. (2024). Investigation of a Novel Atmospheric Pressure Microwave Cold Plasma Torch and Its Characteristics. Chem. Res. Chin. Univ..

[26] Ogata K., Terashima K. (2009). Characterizations of Strip-Line Microwave Micro Atmospheric Plasma and Its Application to Neutralization. J. Appl. Phys..

[27] Rincón R., Muñoz J., Morales-Calero F.J., Orejas J., Calzada M.D. (2021). Assessment of Two Atmospheric-Pressure Microwave Plasma Sources for H_2_ Production from Ethanol Decomposition. Appl. Energy.

[28] Gines A.R.B., Wada M. (2020). Sheet Plasma Excitation by a 2.45 GHz Microwave Power Introduced through a Local Gas Injection Type Dielectric Window. Plasma Fusion Res..

[29] Hamdan A., Liu J.-L., Cha M.S. (2018). Microwave Plasma Jet in Water: Characterization and Feasibility to Wastewater Treatment. Plasma Chem. Plasma Process..

[30] Lebedev Y.A. (2010). Microwave Discharges: Generation and Diagnostics. J. Phys. Conf. Ser..

[31] Ferreira C.M., Moisan M. (2013). Microwave Discharges: Fundamentals and Applications.

[32] Conrads H., Schmidt M. (2000). Plasma Generation and Plasma Sources. Plasma Sources Sci. Technol..

[33] Jin Q., Zhu C., Border M.W., Hieftje G.M. (1991). A Microwave Plasma Torch Assembly for Atomic Emission Spectrometry. Spectrochim. Acta Part B At. Spectrosc..

[34] Bilgic A.M., Prokisch C., Broekaert J.A.C., Voges E. (1998). Design and Modelling of a Modified 2.45 GHz Coaxial Plasma Torch for Atomic Spectrometry. Spectrochim. Acta Part B At. Spectrosc..

[35] Czylkowski D., Hrycak B., Jasiński M., Dors M., Mizeraczyk J. (2013). Atmospheric Pressure Microwave Microplasma Microorganism Deactivation. Surf. Coat. Technol..

[36] Hrycak B., Jasiński M., Mizeraczyk J. (2010). Spectroscopic Investigations of Microwave Microplasmas in Various Gases at Atmospheric Pressure. Eur. Phys. J. D.

[37] Janča J., Klíma M., Slaviček P., Zajíčková L. (1999). HF Plasma Pencil—New Source for Plasma Surface Processing. Surf. Coat. Technol..

[38] Barekzi N., Laroussi M. (2012). Dose-Dependent Killing of Leukemia Cells by Low-Temperature Plasma. J. Phys. D Appl. Phys..

[39] Winter J., Brandenburg R., Weltmann K.-D. (2015). Atmospheric Pressure Plasma Jets: An Overview of Devices and New Directions. Plasma Sources Sci. Technol..

[40] Mizeraczyk J., Dors M., Jasiński M., Hrycak B., Czylkowski D. (2013). Atmospheric Pressure Low-Power Microwave Microplasma Source for Deactivation of Microorganisms. Eur. Phys. J. Appl. Phys..

[41] Haryński Ł., Czylkowski D., Hrycak B., Karczewski J., Gumieniak J., Kramek A., Ryl J., Grochowska K., Dors M., Siuzdak K. (2023). Nitrogen Plasma-Induced Crystallization of Anodic TiO_2_ Nanotubes for Solar Photoelectrochemistry. Appl. Surf. Sci..

[42] Moisan M., Levif P., Nowakowska H. (2019). Space-Wave (Antenna) Radiation from the Wave Launcher (Surfatron) before the Development of the Plasma Column Sustained by the EM Surface Wave: A Source of Microwave Power Loss. AMPERE Newsl..

[43] Nowakowska H., Czylkowski D., Hrycak B., Jasinski M. (2021). Numerical and Experimental Analysis of Radiation from a Microwave Plasma Source of the TIAGO Type. Plasma Sources Sci. Technol..

[44] Sadeghikia F., Talafi Noghani M., Simard M.R. (2016). Experimental Study on the Surface Wave Driven Plasma Antenna. AEU-Int. J. Electron. Commun..

[45] Djourelova M., Petrova T., Ghanashev I., Zhelyazkov I. (1993). Axial Structure of a Shielded Plasma Column Sustained by a Dipolar Electromagnetic Wave. J. Phys. D Appl. Phys..

[46] The COMSOL® Software Product Suite. https://www.comsol.com/products.

[47] Griem H.R., Kolb A.C., Shen K.Y. (1962). Stark Profile Calculations for the Hβ Line of Hydrogen. Astrophys. J..

[48] Gigosos M.A., Cardeñoso V. (1996). New Plasma Diagnosis Tables of Hydrogen Stark Broadening Including Ion Dynamics. J. Phys. B At. Mol. Opt. Phys..

[49] Aliev Y.M., Schlüter H., Shivarova A. (2000). Guided-Wave-Produced Plasmas.

[50] Bogachev N.N., Gusein-zade N.G., Nefedov V.I. (2019). Radiation Pattern and Radiation Spectrum of the Plasma Asymmetrical Dipole Antenna. Plasma Phys. Rep..

[51] Belyaev B.A., Leksikov A.A., Leksikov A.A., Serzhantov A.M., Bal’va Y.F. (2014). Nonlinear Behavior of Plasma Antenna Vibrator. IEEE Trans. Plasma Sci..

[52] Shivarova A., Stoychev T. (1980). Harmonic Surface Wave Propagation in Plasma. I. Second Order Harmonic Waves Generated by One Fundamental Wave. Plasma Phys..

[53] Georgieva V., Berthelot A., Silva T., Kolev S., Graef W., Britun N., Chen G., van der Mullen J., Godfroid T., Mihailova D. (2017). Understanding Microwave Surface-Wave Sustained Plasmas at Intermediate Pressure by 2D Modeling and Experiments. Plasma Process. Polym..

[54] Balanis C.A. (2005). Antenna Theory: Analysis and Design.

[55] (2010). COMSOL RF Module User’s Guide (Ver. 4.1).

[56] Hagelaar G.J.M., Pitchford L.C. (2005). Solving the Boltzmann Equation to Obtain Electron Transport Coefficients and Rate Coefficients for Fluid Models. Plasma Sources Sci. Technol..

[57] Phelps A.V. (1997). PHELPS Database. www.lxcat.net/Phelps.

[58] Pepper D.W., Heinrich J.C. (2017). The Finite Element Method: Basic Concepts and Applications with MATLAB^®^, MAPLE, and COMSOL.

[59] Chen C., Fu W., Zhang C., Lu D., Han M., Yan Y. (2021). Dual-Frequency Microwave Plasma Source Based on Microwave Coaxial Transmission Line. Appl. Sci..

[60] Guan C., Zhan L., Yao S. (2022). Finite Element Simulation and Experimental Research on Uniformity Regulation of Microwave Heating of Composite Materials. Polymers.

