# Supplementary material for: Microwave Plasma Pencil for Surface Treatment: Numerical Study of Electromagnetic Radiation and Experimental Verification
Source: Materials (Basel). 2024 Sep 4;17(17):4369. doi: 10.3390/ma17174369 (PMC11395898; doi:10.3390/ma17174369)
Supplement: Supplementary file 1 [file materials-17-04369-s001.zip › materials-3160589-supplementary.pdf]

The file contains suplementary data to the article

Microwave Plasma Pencil for Surface Treatment: Numerical Study of Electromagnetic Radiation and Experimental Verification.

by: Helena Nowakowska *, Dariusz Czylkowski, Bartosz Hrycak and Mariusz Jasiński

1. Summary of assumptions used in the calculations (described in the text)

- Axial symmetry of both the system and the solutions
- All materials (except plasma) are lossless
- Plasma has the shape of a cylinder rounded at the ends
- Radial distribution of plasma electron density is constant
- Axial distribution of electron density is as in Figure 5
- Gas temperature (and therefore normalized collision frequency, *s*) is constant inside the plasma column
- Plasma permittivity can be determined from the Drude-Lorentz formula
- All media are non-magnetic
- Radius of the radiation sphere is large enough to be in the far field
- There are no other objects in the vicinity of the MPP

2. Mesh used for calculations

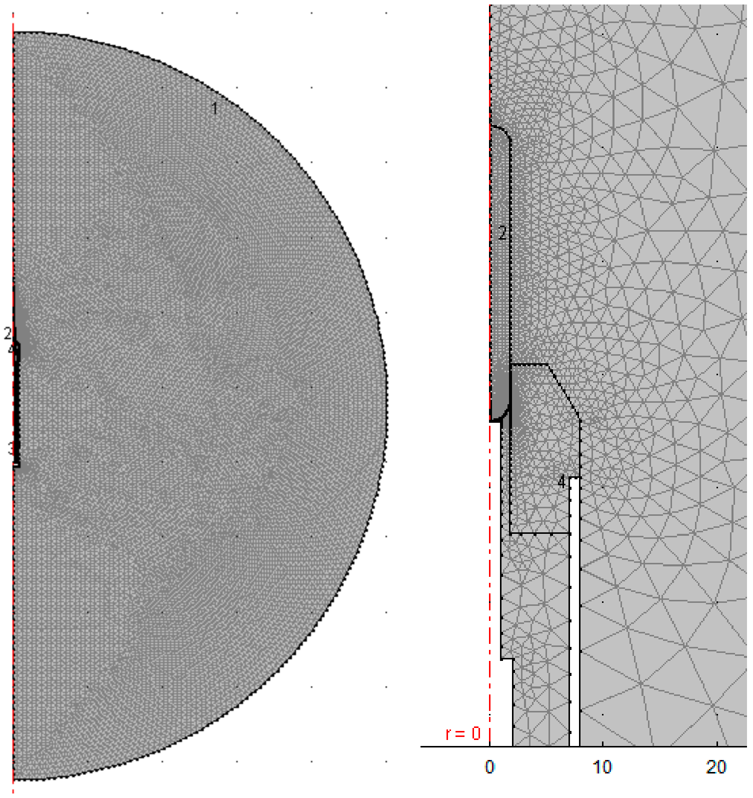

(a) Mesh in the radiation sphere.
(b) Mesh near the plasma

Complete mesh consists of about 22k elements (For R_s = 500mm)

3. Figure 9(a) with changed scale

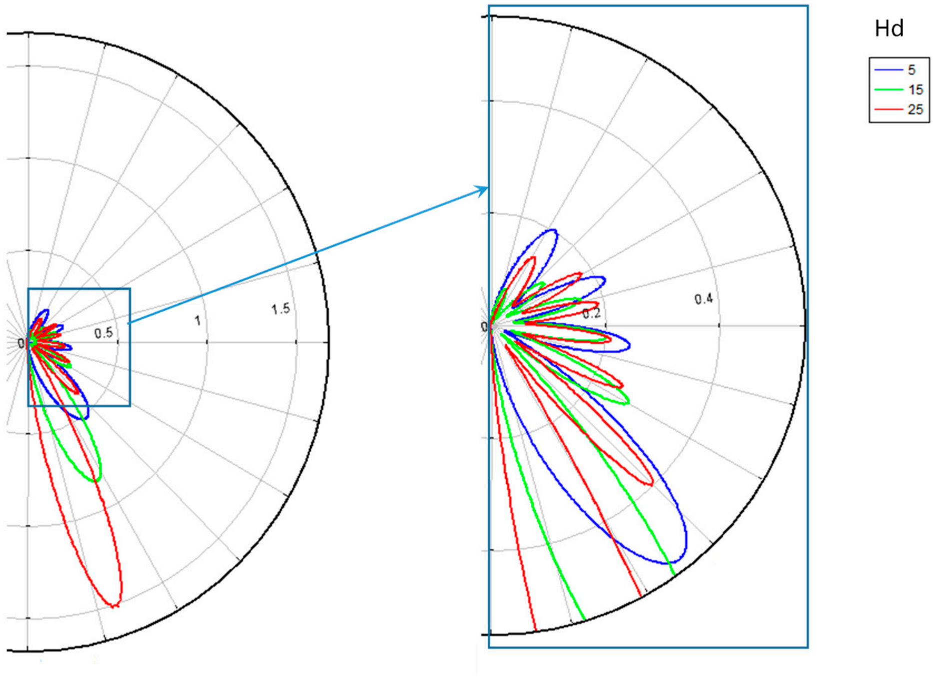

Figure S1. The effect of the H_d on (a) radiation patterns (no cone)

4. Figure 12(a) with changed scale

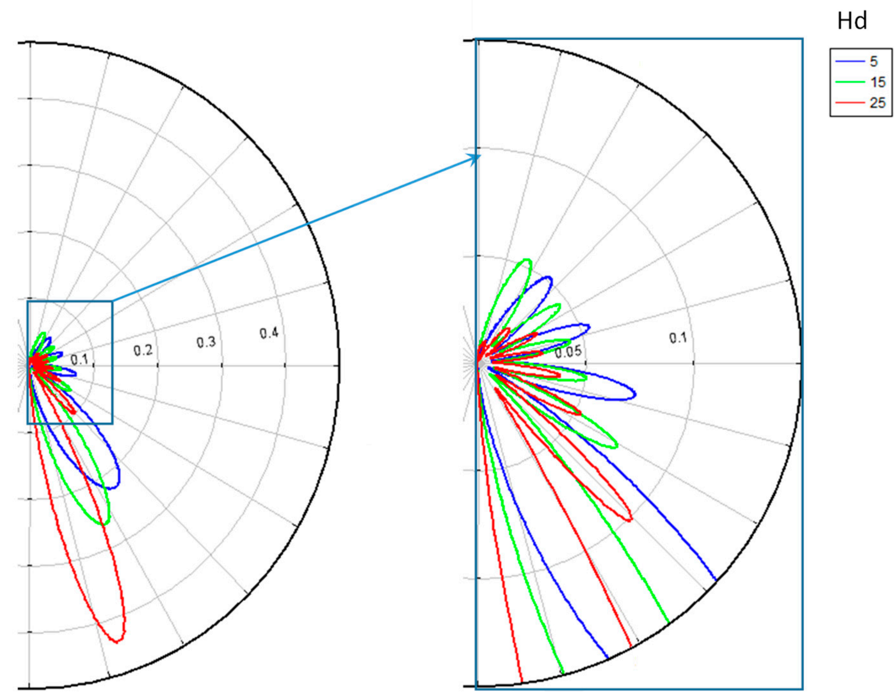

Figure S2. The effect of the H_d on (a) radiation patterns (with cone)
